# Improving Adherence to Physical Therapy in the Development of Serious Games: Conceptual Framework Design Study

**DOI:** 10.2196/39838

**Published:** 2023-11-10

**Authors:** Jorge Fernando Ambros-Antemate, María del Pilar Beristain-Colorado, Marciano Vargas-Treviño, Jaime Gutiérrez-Gutiérrez, Pedro Antonio Hernández-Cruz, Itandehui Belem Gallegos-Velasco, Adriana Moreno-Rodríguez

**Affiliations:** 1 Facultad de Medicina y Cirugía Universidad Autónoma “Benito Juárez” de Oaxaca Oaxaca de Juárez Mexico; 2 Facultad de Sistemas Biológicos e Innovación Tecnológica Universidad Autónoma “Benito Juárez” de Oaxaca Oaxaca de Juárez Mexico; 3 Laboratorio de genómica y proteómica, Centro de investigación Universidad Nacional Autónoma de México-Universidad Autónoma "Benito Juárez" de Oaxaca Facultad de Medicina y Cirugía Universidad Autónoma “Benito Juárez” de Oaxaca Oaxaca de Juárez Mexico; 4 Facultad de Ciencias Químicas Universidad Autónoma “Benito Juárez” de Oaxaca Oaxaca de Juárez Mexico

**Keywords:** conceptual framework, serious game, Flow Theory, adherence, gamification, physical rehabilitation

## Abstract

**Background:**

Insufficient levels of treatment adherence can have adverse effects on the outcomes of physical rehabilitation. To address this issue, alternative approaches to traditional therapies, such as serious games, have been designed to enhance adherence. Nevertheless, there remain gaps in the development of serious games concerning the effective implementation of motivation, engagement, and the enhancement of treatment adherence.

**Objective:**

This study aims to design a conceptual framework for the development of serious games that incorporate essential adherence factors to enhance patient compliance with physical rehabilitation programs.

**Methods:**

We formulated a conceptual framework using iterative techniques inspired by a conceptual framework analysis. Initially, we conducted a comprehensive literature review, concentrating on the critical adherence factors in physical rehabilitation. Subsequently, we identified, categorized, integrated, and synthesized the concepts derived from the literature review to construct the conceptual framework.

**Results:**

The framework resembles a road map, comprising 3 distinct phases. In the initial phase, the patient’s characteristics are identified through an initial exploration. The second phase involves the development of a serious game, with a focus on enhancing treatment adherence by integrating the key adherence factors identified. The third phase revolves around the evaluation of the serious game. These phases are underpinned by 2 overarching themes, namely, a user-centered design and the GameFlow model.

**Conclusions:**

The conceptual framework offers a detailed, step-by-step guide for creating serious games that incorporate essential adherence factors, thereby contributing to improved adherence in the physical rehabilitation process. To establish its validity, further evaluations of this framework across various physical rehabilitation programs and user groups are necessary.

## Introduction

### Background

Physical rehabilitation is widely used in people who have had motor impairment. A rehabilitation model generally consists of performing exercises repetitively with the validation and assistance of a patient-centered team [1]. However, one of the main problems related to rehabilitation is that therapy sessions can be unmotivating or boring when repeating the same exercise, causing discouragement, boredom, depression, or anxiety [2,3]. It can lead to a problem known as “barrier to adherence” [4], which can have a negative impact on outcomes and health care costs. The barrier to adherence can be observed as forgetting, omitting, or incorrectly following medical advice and has implications for treatment cost and efficacy [5]. Studies have shown a high percentage of nonadherence to treatment related to physical activity [6].

Theories and models from different disciplines have been proposed to improve adherence to exercise and reduce the problems described before [7]. For example, a study used psychological intervention in conjunction with physical treatment to change behavior, increase adherence to the recommendations of health personnel, and improve the expected results [8]. Other studies have used technological elements such as video games and virtual reality to improve adherence to an exercise program [9,10].

An auxiliary alternative to conventional therapy is serious games, which are video games created to entertain and achieve at least one additional learning objective [11]. These types of video games also help therapists in supporting patient engagement and motivation and gathering quantitative measures of rehabilitation treatment progression. This type of video game is widely used in various areas, including education, health, behavioral changes, and rehabilitation, among others [12,13].

However, although many studies describe a systematic process for the development of serious games [14-16], they do not consider key aspects of adherence promotion. Besides, the studies were more focused on establishing phases or stages for video game construction; therefore, developers must implement various strategies in serious games that encourage the patients to comply with their treatment.

### Objective

Previous literature has suggested that interventions should incorporate multiple strategies applied simultaneously to enhance exercise adherence, rather than relying on single-strategy interventions [17]. Therefore, this research aims to contribute to existing knowledge by proposing a conceptual framework for the development of serious games. This framework applies theories and strategies rooted in key adherence factors in physical rehabilitation, a user-centered design (UCD) approach, and the GameFlow model, all aimed at improving adherence to physical therapy.

### Key Concepts of Flow Theory, GameFlow, Adherence, and User-Centered Design

#### Overview

This section introduces the components of Flow Theory, the GameFlow model, adherence, the connection between adherence and Flow Theory in the context of physical exercise, and UCD.

#### Flow Theory

Flow Theory, a concept within positive psychology [18], was developed by Csikszentmihalyi through interviews and questionnaires with various individuals, including chess players, rock climbers, dancers, and amateur athletes. These individuals exhibited deep engagement and devotion to their activities, reporting feelings of exhilaration, intense focus, and effortlessness. They did not perceive their experiences as time wasted. Flow, as outlined by Csikszentmihalyi, is considered a fundamental element of enjoyment [18]. He identified 8 common elements in the flow experience, as detailed in Textbox 1.

**Flow elements.**

1. Tasks with the opportunity to be completed
2. Concentration on the task
3. Tasks have unambiguous goals
4. Immediate feedback is provided
5. The person is totally immersed in the task
6. The person has a sense of control over his or her actions
7. Self-concern disappears
8. The person is unaware of the passage of time

#### GameFlow Model

GameFlow [19] is a model that offers criteria for making video games enjoyable and fun. This model is based on Csikszentmihalyi’s Flow Theory [20] and integrates usability and user experience criteria. The GameFlow model consists of 8 elements. The first 7 elements correspond to the Flow Theory: concentration, challenge, player skill, control, clear goals, feedback, and immersion. Concentration is essential to stay focused on video game tasks. Challenges must be included according to the player’s abilities. Player skills encourage the development of skills. The player must perceive control over the activities and clear goals must be described. Further, adequate feedback must be received at the appropriate time. Immersion helps the player feel attracted to the game by forgetting their surroundings. Social interaction includes communication between players. The latter element does not correspond to the Flow Theory, but the authors consider it fundamental to enjoy the games.

Video games developed with the Flow Theory aim to keep players engaged by providing a fun challenge, clear objectives, and continuous progress feedback. This approach creates a sense of control and immersion, enabling players to escape from the worries of everyday life and, as a result, lose track of time while playing.

#### Adherence

Adherence is defined as “the extent to which a patient’s behavior matches the recommendations of the health service provider” [21]. The medical term *instructions* encompasses a range of activities, including taking medication, exercising, and following other recommendations from health care providers [22]. It is crucial to identify adherence factors in patients as this plays a vital role in successfully completing an exercise program.

Some randomized trials of exercise interventions have provided evidence suggesting that exercise can offer mortality benefits similar to various pharmacological interventions in the context of secondary prevention of coronary heart disease, stroke rehabilitation, and diabetes prevention [23]. The adherence to exercise regimens serves as a predictor of long-term efficacy in exercise therapy, leading to reduced pain and improved physical function [24], and it is a central component of injury rehabilitation [25].

#### Adherence to Physical Activity and Flow

Promoting treatment adherence presents a challenge across various health domains [26]. Elbe et al [27] demonstrated that individuals participating in a randomized exercise intervention reported a high level of *flow*, an intrinsically motivating state that can positively impact long-term adherence. Petosa and Holtz [28] aimed to test the Flow Theory of exercise adherence and discovered that intrinsic motivation plays a crucial role in sustaining adherence to physical activities. Schüler and Brunner [29] proposed that experiencing flow during physical activities can enhance long-term adherence, as individuals find the activity rewarding and enjoyable, increasing the likelihood of regular participation.

Csikszentmihalyi et al [30] contended that individuals experience a highly intrinsically motivated state of flow when they are appropriately challenged by their tasks, and their skills are well-balanced. Maintaining individuals in a state of flow during physical rehabilitation promotes better long-term adherence to treatment [31].

#### User-Centered Design

UCD takes various definitions, but all share a common emphasis on addressing user needs throughout the design process, ultimately aiming to create products that are usable and understandable [32]. A UCD approach typically involves 3 key phases: analysis, design, and evaluation [33]. Integrating UCD into software development processes ensures that user experience is a core consideration from the project’s inception, leading to a highly usable end product.

## Methods

### Conceptual Framework Development

#### Overview

**Figure 1:**
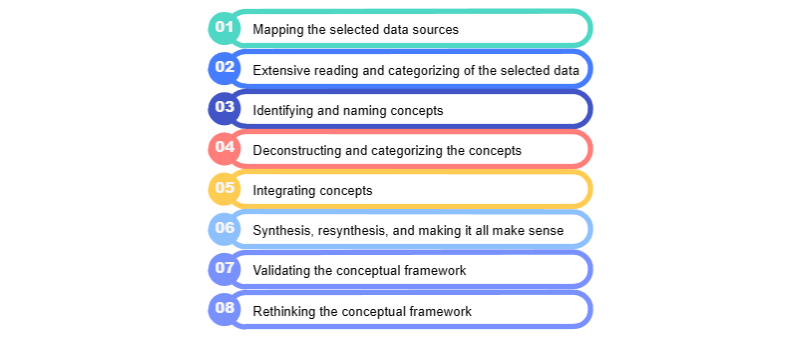
The 8 phases of the methodology by Jabareen.

A conceptual framework is a network of interconnected concepts designed to provide a comprehensive understanding of the phenomenon of interest [34]. Our development process for the conceptual framework is based on iterative analysis techniques, encompassing 8 phases as outlined by Jabareen [35], which is illustrated in Figure 1.

In this study, the development of the conceptual framework will be condensed into 6 stages. The phases of “Validating the Conceptual Framework” and “Rethinking the Conceptual Framework” are aspects that we anticipate exploring in future research. The following sections will provide a detailed description of the implementation of each of the aforementioned phases.

#### Phase 1: Mapping the Selected Data Sources

In the first phase, the process commenced with a comprehensive review of multidisciplinary texts. To conduct this research, we adhered to the recommendations outlined by Templier and Paré [36] and Cant et al [37]. The literature review is presented in Multimedia Appendix 1 (also see [38-52]).

#### Phase 2: Extensive Reading and Categorizing of the Selected Data and Phase 3: Identifying and Naming Concepts

During these phases, key adherence factors were extracted from the “Results” and “Discussion” sections of each paper in the reviews. Subsequently, the information obtained through data extraction underwent a comparative analysis by 2 reviewers (JFAA and MPB-C). In instances of disagreement, the reviewers engaged in discussions until a consensus was reached. The identified key adherence factors can be found in Multimedia Appendix 2 (also see [38-52]).

#### Phase 4: Deconstructing and Categorizing the Concepts

In the fourth phase, the goal was to organize and categorize the concepts based on their characteristics or attributes. As many concepts serve similar purposes, this phase involved categorizing them accordingly.

#### Phase 5: Integrating Concepts

The objective of the fifth phase was to significantly reduce the number of concepts, making it manageable for manipulation. The concept integration is presented in Multimedia Appendix 3.

#### Phase 6: Synthesis, Resynthesis, and Making It All Make Sense

In the sixth phase, the objective was to create a diagram or model illustrating the relationships between the identified concepts. Following this, the team proceeded to integrate these concepts into the serious game development process. This involved conducting a series of analyses on existing theories related to product/software development. After careful analysis, the team identified 2 cross-cutting themes: UCD [33] and the GameFlow Model. UCD principles focus on the development of engaging products that encourage user adoption, taking into account user characteristics and needs right from the beginning of the development. Another model, the GameFlow model [19], was used to infuse more enjoyment into video games and to integrate the principles of Flow Theory into the development process. This theory has been found to enhance treatment adherence [31].

### Ethics Approval

The information used in this study was collected from previous studies with unidentified data and without direct access to the participants. For this reason, this research is not considered to involve human participants.

## Results

### Conceptual Framework Proposal: Literature Search and Review

The literature search yielded a total of 15 studies [38-52], all of which were reviews reporting key adherence factors in physical rehabilitation. The results from these reviews are detailed in Multimedia Appendices 1-3 (also see [38-52]).

The framework is structured as a road map, illustrating the sequential development of a serious game. It consists of 3 main phases: understanding context, development, and evaluation. These phases align with the principles of analysis, design, and evaluation in the UCD approach [33,53].

**Figure 2:**
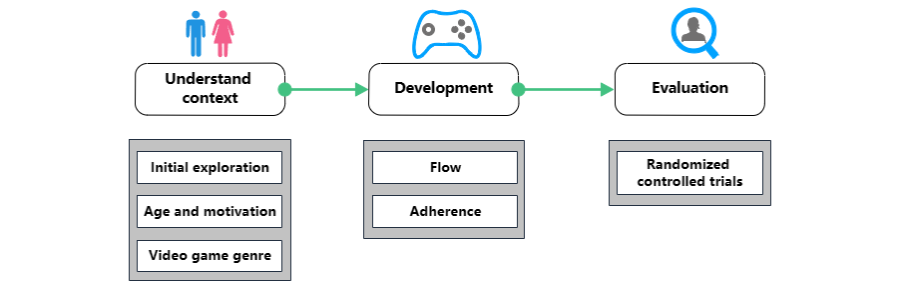
Conceptual framework.

Figure 2 displays the conceptual framework, and we will now explore the individual elements that constitute this framework, along with their specific attributes.

### Understand Context Phase

#### Overview

The first phase consisted of 3 interlinked steps aimed at identifying the characteristics of target users, as well as differences related to age and video game genre preferences.

**Figure 3:**
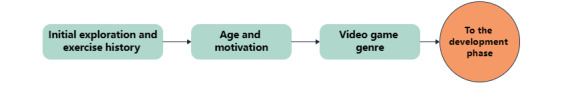
The process to understand the context.

In the first step, it is essential to consider the diverse characteristics of the population when creating a product or service. Individuals vary in terms of physical, social, psychological, and technical conditions. Understanding and considering these differences in a system offer numerous advantages, including broader adoption, cost savings, and the development of safer and more efficient systems [54]. It is vital to involve users’ perspectives from the outset, as this is crucial for accurately identifying and categorizing their needs and expectations, ultimately transforming them into achievable and specific requirements. In the second step, developing serious games necessitates the recognition of age-related preferences for different types of video games. In the third step, the video game genre should be taken into account to enhance patient acceptance of the game. Figure 3 illustrates this process.

#### Initial Exploration and Exercise History

Identifying patient requirements and characteristics is a crucial step before commencing treatment and designing a serious game. In our review of papers [38-40,46-49], we found that the initial exploration and exercise history were described in 47% (7/15) of the papers. To enhance adherence, it is important to consider elements such as the patient’s health status, prior therapies, and habits. In health care, a multidisciplinary team must assess patients’ physical and mental characteristics as these factors can significantly impact adherence. For instance, a patient with a chronic condition that causes pain may have reduced adherence to rehabilitation exercises [55]. It is the responsibility of health care professionals to prescribe treatments that alleviate or eliminate such pain. Additionally, evaluating the patient’s history of previous therapies and identifying habits, such as smoking, alcohol consumption, and exercise routines, can help determine the most effective approach to enhance adherence to new exercises.

An initial examination by health care professionals is essential. This examination allows for a thorough assessment of the patient’s health status, aiming to identify potential barriers or facilitators to treatment adherence. Consequently, it enables the establishment of personalized treatments and exercise regimens with appropriate intensity, duration, frequency, and exercise types, ultimately improving adherence.

#### Age and Motivation

Age often influences an individual’s preference for specific types of games. When patients feel comfortable using a video game, they are more likely to enter a state of flow, which, in turn, promotes usage and enhances adherence. Several studies have explored how age can impact motivation and overall experience [56]. Hence, it is essential to consider age during the planning and development of a serious game. Children tend to be more motivated by games featuring elements of fantasy, socialization, and a balance of fun and challenge [57]. Conversely, teenagers and adults generally prefer games with competitive elements, aiming to become the top player and overcome challenging levels. Additionally, as children mature into teenagers, their interest often shifts toward social interaction through online games, including e-sports and massively multiplayer online role-playing games [58]. Lastly, it is important to note that many older adults may experience a decline in processing speed, cognitive functions, and reflexes [59]. Consequently, it is advisable to recommend puzzles or casual video games [60] as they are characterized by simple rules and do not require special skills to play.

#### Video Game Genre

Serious games should align with individuals’ gaming preferences and expectations. Everyone has their own affinity for specific video game genres, which can lead to enjoyment and, subsequently, bring about behavioral changes that foster game adherence with increased enthusiasm and frequency. Video games are classified into various genres based on their gameplay and style. Lucas and Sherry [61], drawing from sources such as video game manufacturers, gaming websites, and gaming magazines, outlined a classification that includes genres such as Strategy, Fantasy/Role-Playing Games, Adventure, First-Person Shooter, Fighting, Simulation, Classic Arcade Games, Card/Dice Games, Quiz/Trivia, Board Games, Kids Games, Sports, Racing/Speed, and Puzzles. Research has indicated that genre preferences may also be influenced by an individual’s gender [62]. Typically, men tend to gravitate toward genres such as Sports, Role-Playing Games, First-Person Shooter, and Adventure games, while women often favor Racing/Speed, Puzzles, and Adventure games. Interestingly, studies have demonstrated that both men and women share a common preference for Adventure games.

### Development Phase

#### Overview

Building upon the groundwork laid in the “understand context” phase, which involves identifying the patient’s requirements, characteristics, and selecting an appropriate game type through initial exploration, the phase of constructing the serious game commences. The development phase is focused on integrating elements derived from key adherence factors and the GameFlow model into the video game.

Incorporating elements from the original GameFlow model, this framework utilizes elements such as concentration, feedback, immersion, and social interaction. As for challenge/player skill and control, some authors argue against separating these elements [63,64], so they have been integrated into the concept of adaptivity. Within this framework, adaptivity is intertwined with maintaining a delicate equilibrium between the patient’s challenge and skill levels, which is especially crucial for those with physical limitations. A gradual progression of challenge is essential, allowing the patient to maintain a sense of control and receive personalized exercises tailored to their specific limitations.

From the key adherence factors, accessibility is integrated with usability. This combination is based on studies that have emphasized the interdependence of these 2 concepts when implementing an interactive system, rather than treating them as isolated elements [65].

**Figure 4:**
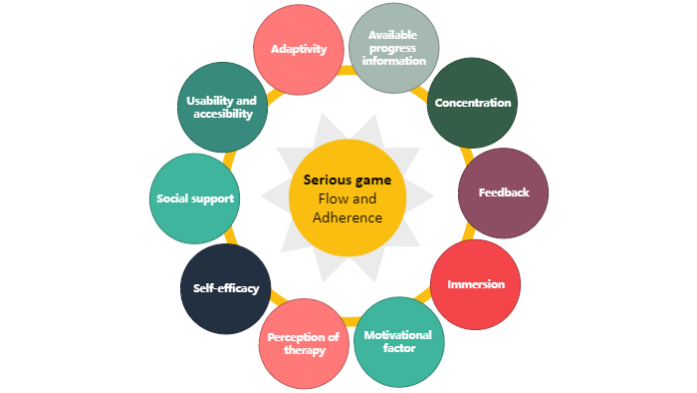
Key adherence factors and GameFlow-based elements in the development phase.

By combining the principles of the GameFlow model with key adherence factors in the development of the serious game, we establish a foundation for crafting an enjoyable video game that not only motivates patients to sustain its use but also bolsters treatment adherence. Figure 4 illustrates the key components of this phase.

#### Adaptivity

Developing adaptive games offers the opportunity to tailor exercise programs to better align with patients’ limitations. Personalized exercise programs, encompassing aspects such as exercise type, duration, frequency, and intensity, play a crucial role in promoting adherence. Such personalization enhances the patient’s perception of progress in their rehabilitation treatment. It is noteworthy that in 60% (9/15) of the papers in our review, the concept of a personalized exercise program was discussed [39,41-44,48,49,51,52]. Maintaining a balance between challenge and skill, while adjusting difficulty based on the player’s needs, is essential to ensure a positive gaming experience, prolong usage, and sustain motivation.

Research has indicated that an appropriate level of challenge relative to the player’s skills fosters improved acceptance, commitment, motivation, and learning, ultimately leading to prolonged use [66].

Achieving an optimal immersion experience, deep enjoyment, and heightened engagement relies on maintaining a balance between an individual’s abilities and the challenges they face. When the challenge is not sufficiently difficult, it can lead to feelings of boredom or detachment. Conversely, when the challenge surpasses an individual’s ability, it can lead to anxiety, stress, and ultimately, resignation. People’s abilities and affective states evolve over time as they become proficient in the game. If a serious game system for physical rehabilitation does not incorporate adaptability in its challenge levels, patients are at a higher risk of becoming disengaged, experiencing boredom, and ultimately discontinuing their treatment [67].

An adaptive game can effectively manage challenge levels for each individual, preventing players from becoming stuck, tailoring the game to their preferences and requirements, and even identifying when the game is no longer providing an adequate level of challenge.

Incorporating adaptability into a game involves increasing the difficulty level in line with the player’s skill. A common approach to achieving this is by including an option to manually adjust the difficulty, which clinicians can use to tailor the game’s challenge to the patient’s abilities. Various techniques are utilized, such as those based on scoring systems [67], procedural content generation and semantic modeling [68], genetic algorithms [69], and artificial intelligence [70]. An effective adaptive system should possess the capability to analyze the patient’s skill information and draw inferences from the data collected. This allows the patient to adjust the game’s challenge level, providing the player with a sense of control.

#### Available Progress Information

In our review, progress information was found in only 33% (5/15) of the papers [39,43,45,48,49]. One of the initial steps in enhancing adherence is to determine whether the treatment is being correctly followed. Often, physicians may have limited information regarding treatment adherence. In many cases, physicians rely solely on the information provided by the patient [26]. However, this approach can be problematic, especially in situations where rehabilitation is slow and gradual. It is crucial for patients to receive objective data that keep them informed about their progress and help them understand the changes and improvements they are experiencing. Recognizing the benefits attained through exercise is vital for sustaining engagement and enhancing adherence. To facilitate this, serious games can offer accessible and comprehensible reports on the rehabilitation process. These reports may include graphical representations and statistics, as the inclusion of objective measures serves to motivate both patients and medical professionals to persist with the treatment.

#### Concentration

The games should engender intense concentration or complete absorption. As Csikszentmihalyi [20] has highlighted, concentration is a fundamental element for achieving a state of flow. Prior research has demonstrated that maintaining the patient’s concentration on the game can redirect their focus away from their medical treatment, the ailment they are contending with, and in certain instances, their pain. This diversion contributes to improved adherence to rehabilitation exercises [38,71]. The game should capture attention from the outset and sustain it throughout the gaming experience. High concentration helps in diverting attention away from external stimuli, ensuring a consistent focus [72]. To foster concentration in a video game, it is important to avoid overloading the game scene with distractions. For instance, this could involve limiting the number of nonplayer characters, moving objects, vehicles, obstacles, and other elements [73]. Additionally, clear task objectives are essential to reduce negative emotions and foster positive ones.

#### Feedback

A common element found in all 15 of the papers in our review [38-52] was feedback. Feedback is crucial for learning, and as noted by Pritchard et al [74], it has a positive impact on performance. Feedback serves as a valuable guide to help individuals achieve specific goals. It plays an essential role in instructional design, enhancing and sustaining cognitive engagement, and ultimately contributing to treatment adherence. Feedback constitutes a fundamental interaction between the game and the user, distinguishing between the expected and actual performance. It can be implemented through various modalities, including visual, auditory, haptic, or thermal options. Visual feedback can be delivered through messages or avatars that assist in correcting improper movements. Additionally, visual feedback may take the form of scoring, rewards, achievements, and experience points [75]. Auditory feedback is conveyed through elements such as voices, sound effects, and variations in background music [76]. Haptic feedback is typically integrated through an interactive interface, utilizing servomotors with varying intensity levels to inform the patient about the correctness of their exercise performance [77]. By contrast, while less commonly used, thermal feedback has the potential to convey emotions to individuals [78].

The utilization of technology, particularly through wearable systems, proves instrumental in monitoring exercises, collecting posture information, and tracking limb movements, thereby bolstering treatment adherence [79]. Wang et al [79] conducted a systematic review to identify and categorize interactive wearable systems designed for monitoring movement and posture during upper-body rehabilitation. Commonly, accelerometers and inertial measurement units were used to monitor and offer feedback on movement performance during rehabilitation. Furthermore, the use of smartphones for monitoring physical activity and providing feedback is on the rise. As noted by Collado-Mateo et al [39], technology offers precise control over physical activity, allowing for the customization of exercise plans in terms of frequency, duration, and timing. It also enables real-time feedback, reminders, communication between patients and health care professionals, and the sharing of activities with colleagues, among other advantages.

Lastly, gathering user feedback on the visual and operational aspects of the video game is crucial for enhancing the interactive system. This feedback can be collected in written formats, including questionnaires and comments, as well as through verbal formats, such as interviews.

#### Immersion

Immersion involves a state of being unaware of time and the real world, providing a distraction from daily concerns. This state is also considered crucial for truly enjoying the game [80]. A pleasurable experience when using a video game lies in its capacity to induce immersive experiences. Therefore, the incorporation of immersion is key to creating an environment where the player feels immersed in the video game. Research suggests that video games featuring immersive elements lead to heightened enjoyment and improved adherence to the intervention regimen [81].

In the development of the serious game, the quest for enhanced immersion necessitates an environment devoid of external distractions or an overabundance of environmental elements [82]. Both the story (the events within the virtual world) and the narrative (how these events are structured and sequenced) are vital elements in achieving this immersion. These elements, when used effectively, evoke empathy and foster emotional engagement among users [83]. Audio is another crucial aspect to consider in achieving immersion; sound effects and music are also used in occupational therapy to assist patients in exercising joints and muscles [84]. Music, in particular, stimulates the brain areas related to emotional processing and motor control [85]. Research has demonstrated that background music and sound effects in video games contribute to immersion and have the potential to alleviate tension [86]. However, it is important that background music is not chosen randomly in a serious game. It should be thoughtfully integrated to establish a connection with other elements, such as the environment and the storyline, to resonate with the user. Furthermore, audio elements such as sound effects, ambient sounds, and nonplayer character voices enhance emotional engagement and stimulate the player’s imagination [87]. Therefore, the way sound is perceived can significantly influence the player’s attention and their unconscious emotional state.

#### Motivational Factor

##### Motivation and Maintaining Adherence

Motivation is another recurrent element in the literature, as it was found in all 15 papers in our review [38-52]. Numerous studies have put forth motivational strategies aimed at fostering functional improvement and adherence.

Maintaining adherence to a rehabilitation process over time can pose challenges for both patients and health care providers. Many patients may experience feelings of frustration or depression due to their condition. Therefore, it becomes crucial to incorporate motivational strategies to enhance treatment adherence. Two types of motivation exist: extrinsic and intrinsic [88]. Extrinsic motivation is fueled by external rewards, such as trophies, monetary incentives, or verbal feedback such as praise. By contrast, intrinsic motivation is nurtured by internal rewards, where individuals find something inherently interesting or enjoyable. Motivation can take various forms, including feedback or social support, demonstrating treatment progress through clear objective measurements, self-efficacy, and gamification. While the first 4 elements are integrated into this framework, gamification is explained in the section below.

##### Gamification

Researchers have been exploring the application of game motivation to diverse nongame scenarios, a concept known as “gamification.” Gamification is defined as “the use of game design elements in nongame contexts” [89]. This approach seeks to leverage the motivational aspects of games to enhance user engagement, enthusiasm, perseverance, and achievement. Therefore, the incorporation of gamification is a crucial component in the development of serious games. Research has demonstrated that patients experience enhanced motivation and physical capacity when engaging in tasks that incorporate gamified challenges [90]. Another study revealed that patients experienced increased motivation to engage in their treatments, resulting in improved adherence to exercise, commitment, and social interaction [91]. Some of the most commonly utilized gamification elements that have been shown to have a positive impact on individuals who play video games are points, levels, leaderboards [92], achievements/badges, stories/themes, rewards, and feedback [93].

Utilizing these gamification elements serves to provide extrinsic motivation to the user. Nevertheless, it is important to note that they can also unconsciously stimulate internal motivation. For instance, leaderboards and badges earned through treatment progress can evoke emotions and engage the patient, encouraging them to complete activities satisfactorily and even prompt them to repeat exercises to improve their scores and ascend the leaderboard ranks.

#### Perception of Therapy

The perception of therapy was discussed in 67% (10/15) of the papers included in our review [38-41,44,46-49,52]. It is crucial to inform and remind the patient about the advantages of adhering to the rehabilitation plan and make them aware of the expected exercise outcomes. This approach can significantly boost exercise adherence.

Participants are more likely to engage in and sustain their prescribed exercises when they observe improvements in their health as a direct result of their treatment adherence. Communicating the benefits, objectives, expectations, and even the sensations associated with these exercises is a responsibility shared by health care professionals both in their clinical practice and within the serious game. Health care professionals should exercise caution when discussing gradual changes during therapy to ensure that patients maintain realistic expectations and avoid overly optimistic beliefs [39]. In the serious game, patients should receive information through on-screen messages, images, audio, or video about each exercise they perform in the virtual game and the expected benefits.

#### Self-Efficacy

Self-efficacy, defined as an individual’s confidence in their ability to successfully perform a behavior or achieve an objective [94], is widely recognized as a pivotal element for enhancing adherence. In fact, self-efficacy was a key focus in 73% (11/15) of the papers within our review [38,39,42-47,49,51,52]. This concept holds the power to instigate behavioral change, particularly in individuals undergoing treatment. Self-efficacy has found application in various health domains, including weight loss, nutrition, and physical activity. In the serious game being developed, several elements can be incorporated to boost self-efficacy, such as promoting familiarity with other participants through social support, facilitating communication with staff, optimizing the environment, and providing constructive feedback. Fostering familiarity with other participants in the program connects patients with individuals who share similar experiences, promoting social interaction [39-42,44-49,51]. Establishing familiarity with the staff involves health care professionals providing formal social support, support, and motivation [38-52]. Creating a familiar and supportive environment, which encompasses the facilities, home settings, and materials used, is essential for enabling autonomy and a sense of relatedness. This boosts individuals’ confidence as they perform exercises, especially when supported by devices and in a secure environment [40,41,43-46,48,49]. Finally, offering feedback on the progress toward goals achieved after each rehabilitation session significantly contributes to enhancing patients’ perceived self-efficacy [39].

#### Social Support

##### Overview

Social support was a topic of discussion in all 15 papers included in our review [38-52]. Social interaction is an integral aspect of human existence, as our brains and minds continually evolve through interactions with others [95]. This dynamic interaction encompasses communication, cooperation, imitation, assistance, and negotiation. Social support has been defined as “the assistance and protection a person has through social ties with other people or a community” [95]. Social support has the remarkable ability to reduce stress and boost self-esteem. It can take various forms, including formal support from health care professionals, natural support from family and friends, and community-based support.

##### Formal Social Support

Formal social support is offered by health care professionals, including therapists, doctors, nurses, and other medical personnel engaged in the patient’s rehabilitation process. The significance of this form of support is emphasized by numerous authors. Room et al [42] highlighted that interventions involving feedback and monitoring by health care professionals can effectively address exercise adherence barriers, particularly in older individuals. According to Collado-Mateo et al [39], supervision plays a crucial role in maximizing benefits while reducing the risk of injury, emphasizing the importance of continuous feedback and communication. Farrance et al [40] emphasized the significance of therapist motivation and the inclusion of enjoyable exercises in the program. Lastly, Rodrigues et al [41] recommended a positive attitude from the therapist and regular exercise supervision. In summary, formal social support plays a pivotal role. It not only guides the selection of exercises in the rehabilitation process but also offers essential feedback and monitoring to maximize benefits and prevent injuries. Furthermore, maintaining constant communication is crucial for patient motivation, addressing concerns, and fostering a positive attitude.

Telerehabilitation can serve as a form of formal support in serious games [96]. Leveraging communication technologies enables seamless interaction between health care professionals and the patient. These professionals can offer feedback, monitor progress during sessions using the statistics derived from the serious games, and maintain communication with the patient through messages for upcoming training sessions.

##### Natural Social Support

Natural social support is derived from close relationships, such as family and friends. Research has consistently demonstrated that the presence and collaboration of these individuals significantly enhance the overall gaming experience and the emotional quality elicited during gameplay [38,39,42,48,49,51]. Social interaction holds significance across all age groups, but it particularly stands out in the case of older adults. Encouraging social interaction is recommended to mitigate issues such as mortality or depression resulting from social isolation [97]. A study by Jurkiewicz et al [98] found that 50% of patients with stroke undergoing rehabilitation attributed their motivation to comply with home rehabilitation therapy to the presence of social support. In this form of support, family and friends may offer financial assistance for acquiring necessary equipment or aid patients in carrying out exercises and positioning devices when their disabilities hinder independent execution.

##### Social Support by the Community

Community-based social support pertains to collective groups of individuals undergoing similar rehabilitation processes. Such groups can foster an enjoyable environment [39-42,44-49,51]. Sharing experiences and common interests within these peer communities can enhance a sense of belonging and contribute to the overall enjoyment of a class or activity [99].

##### Application of Natural Support and Community in Serious Games

The incorporation of social interaction in serious games can be facilitated through various communication channels, including face-to-face communication, global groups, subgroups, chats, message exchanges, and forums [100]. Furthermore, social networks can be utilized as a platform for social interaction in at-home activities. Maher et al [101] have identified 2 approaches to online social media–based interventions: interventions that utilize social media websites such as Facebook and Twitter, and independent social networks that are specifically oriented toward health-related topics. Serious games can potentially incorporate elements from both of these approaches to enhance social interaction and support among users. The first approach for natural support involves sharing game progress on social networks. As for the community aspect, participants can respond to their peers’ achievements, including suggestions from friends and leaderboards, within a video game–specific social network [92]. Gotsis et al [102] created a web-based intervention (Wellness Partners) in the form of a physical activity diary integrated into a game with social networking. The social network includes several features, such as posting physical activities or setbacks, sending private messages, reviewing the complete history of updates, viewing a tag cloud displaying the posted physical activities by all members, and exchanging virtual gifts.

#### Usability and Accessibility

Usability and accessibility should be fundamental components of an interactive system [65] and are crucial elements to incorporate into the serious game. Individuals engaged in an intervention regimen using a serious game should have access to user interfaces and data-acquisition devices that are user-friendly and easy to use. Research has suggested that proper interaction with software applications in a rehabilitation treatment leads to improved adherence to the intervention, as individuals feel more confident using the interactive system [103]. Usability pertains to how users use the system to accomplish specific goals with effectiveness, efficiency, satisfaction, and ease [104]. Accessibility encompasses considerations related to designing systems that are accessible to individuals with various physical, sensory, and cognitive abilities [105]. In our review, accessibility was addressed in 60% (9/15) of the papers [40,41,43-49]. If systems prioritize accessibility without considering usability, individuals with disabilities may be able to use the software, but they might not have an enjoyable user experience due to usability errors (eg, a saturation of elements, incorrect placement of text or images, and difficulty to use). Conversely, if a system focuses on usability without considering accessibility, it means that the needs of individuals with disabilities are not taken into account. Methods of usability engineering can be applied to ensure a user-friendly experience [106]. Additionally, established strategies and guidelines are available to incorporate accessibility features into video games [107,108], web-based systems [109], and mobile apps [110].

### Evaluation Phase

A randomized controlled trial is a valuable method for gathering robust evidence on the effectiveness of an intervention in the field of medicine [111]. This evaluation typically involves 2 groups: an experimental group that utilizes the serious game as part of the rehabilitation process, and a control group that receives conventional treatment. Following this, both groups should be tracked over time to assess differences in outcomes. This can be achieved by conducting motor evaluation tests, such as the Wolf Motor Function Test or the Fugl-Meyer Assessment for the upper limb, the Berg Balance Scale for evaluating balance and posture, and the Lower Extremity Motor Coordination Test for the lower limb. The results of the trial and their subsequent analysis will help evaluate the effectiveness of the intervention.

## Discussion

### Principal Findings

In this paper, we introduce a conceptual framework grounded in key adherence factors. We have integrated the UCD approach and the GameFlow model as overarching themes: UCD serves as the foundation for structuring the framework’s phases, while the incorporation of GameFlow model elements is aimed at creating video games that enhance concentration, immersion, and enjoyment. The conceptual framework comprises 3 primary phases. The first phase, termed “Understanding Context,” focuses on discerning user characteristics, needs, and game preferences. In the second phase, the framework entails the development of a serious game that leverages key adherence factors to enhance treatment adherence. Lastly, in the third phase, the serious game is subjected to an evaluation process.

We recommend utilizing this framework in conjunction with a systematic development process to effectively incorporate key adherence factors and flow elements into the construction of serious games, thereby enhancing adherence in the rehabilitation process.

Through this review, we identified 9 common key adherence factors: (1) motivation, (2) social support, (3) feedback, (4) self-efficacy, (5) perception of therapy, (6) personalized exercise program, (7) accessibility, (8) initial exploration and exercise history, and (9) available progress information. The examination of each of these key adherence factors can offer valuable insights for future research directions in this field.

Additionally, this framework can be adapted for the development of serious games aimed at promoting adherence in various other areas. Examples include medication adherence, addiction prevention or reduction, and behavior improvement, such as promoting sun protection or teaching correct teeth-brushing techniques. The principles of this framework can be applied to address a wide range of adherence-related challenges.

### Comparison With Prior Work

The existing literature on the development of serious games has often focused on defining phases or stages for video game construction. For example, the framework proposed by Amengual et al [16] outlines a process for developing serious games specifically designed for motor rehabilitation. These frameworks serve as valuable guides in the development of serious games for targeted purposes. The dimensions proposed are the project initiation activity, an iterative flow composed of 4 development activities (planning, modeling, construction, and evaluation), and a clinical study for validation. While this framework provides a systematic method for video game development, it does not consider elements related to adherence, such as feedback, adaptability, and patient follow-up. Zain et al [112] present a framework based on GameFlow, in which game designers and developers create user interfaces tailored for users with motor impairments to enhance their experience while playing serious games. The framework includes elements such as player skill, challenge, concentration, feedback, immersion, learning opportunities, accessibility, and adaptivity. These elements, which are related to the GameFlow model, are incorporated into the framework to enhance the graphical interfaces of serious games during development. In the context of adherence, the framework primarily focuses on adaptivity, accessibility, and feedback. However, it does not incorporate other strategies, such as gamification elements. In another study, Peñeñory et al [113] proposed a guide for developers of serious games aimed at the psychomotor rehabilitation of children with hearing impairments. The methodology consists of 6 phases: analysis of patients and activities, conceptualization of the game and player experience, design of formal game elements, design of requirements and interaction, design of prototypes, and game testing. Although this methodological proposal is supported by continuous iterative feedback, each phase describes a series of activities for constructing the serious game without explicit incorporation of key adherence factors.

Thus, a conceptual framework was required to guide the development of serious games with key adherence factors. Our framework is unique as it specifically emphasizes the development of serious games with these factors to enhance adherence to physical rehabilitation treatment. It offers clear and practical phases that can be implemented by multidisciplinary teams.

### Study Limitations

While we used techniques and search strategies to identify papers using keywords in the selected databases, it is possible that some relevant studies were unintentionally omitted. We acknowledge the limitations of the conceptual framework, including the need for validation through the collaborative development of a serious game with health experts and subsequent evaluation through a randomized controlled trial.

### Conclusions

Using a combination of strategies in rehabilitation, particularly through the use of serious games, has been shown to enhance exercise adherence and encourage participants to complete rehabilitation programs. The conceptual framework presented here is an attempt to address this need, dividing the process into 3 phases and integrating 2 cross-cutting themes. Rigorous game development that incorporates key adherence factors is essential to ensure better treatment adherence while maintaining an enjoyable gaming experience. To establish the validity of this framework, further evaluation of diverse physical rehabilitation programs involving a variety of users is necessary.

## Appendix

Multimedia Appendix 1 Literature review of papers with key adherence factors in physical rehabilitation.

Multimedia Appendix 2 Key adherence factors identified.

Multimedia Appendix 3 Concept integration.

## Abbreviations

UCD: user-centered design
