# Supplementary material for: Improving Adherence to Physical Therapy in the Development of Serious Games: Conceptual Framework Design Study
Source: JMIR Form Res. 2023 Nov 10;7:e39838. doi: 10.2196/39838 (PMC10674146; doi:10.2196/39838)
Supplement: Multimedia Appendix 1 [file formative_v7i1e39838_app1.pdf]

**Multimedia Appendix 1**

# Literature review of papers with key adherence factors in physical rehabilitation.

We reviewed the literature to identify relevant papers, Google Scholar, IEEE Xplore, ACM Digital Library and PubMed were used for data acquisition. The electronic search was performed in February 2022 and updated in august 2023. In the final search strings, the terms shown in Textbox 1 were used.

Search terms

A term

- A1. physical

B term

- B1. Rehabilitation

C term

- C1. Exercise

D term

- D1. Adherence

E term

- E1. Not cancer
- E2. -cancer

Textbox 1. Search terms for the search string

## Inclusion Criteria

Only studies with the following criteria were considered eligible for inclusion: reviews related to key adherence factors in physical rehabilitation, articles published in English, and all reviews regardless of the year of publication.

## Exclusion Criteria

Articles duplicated, papers regarding opinion pieces, papers that are not related to physical rehabilitation were excluded from the study.

## Study selection

The search retrieved a total of 17,841 registers. Duplicate records were removed using the software Mendeley Reference Manager. Studies that met the eligibility criteria by reading the title and abstract were retrieved in full text. Any disagreement was addressed by another reviewer who made the final decision. After that 15 studies were included. The selection process is summarized in the Preferred Reporting Items for Systematic Reviews and Meta-Analyses (PRISMA) flow diagram (figure 1). Table 1 shows the number of documents retrieved from each database.

Figure 1. PRISMA flow diagram

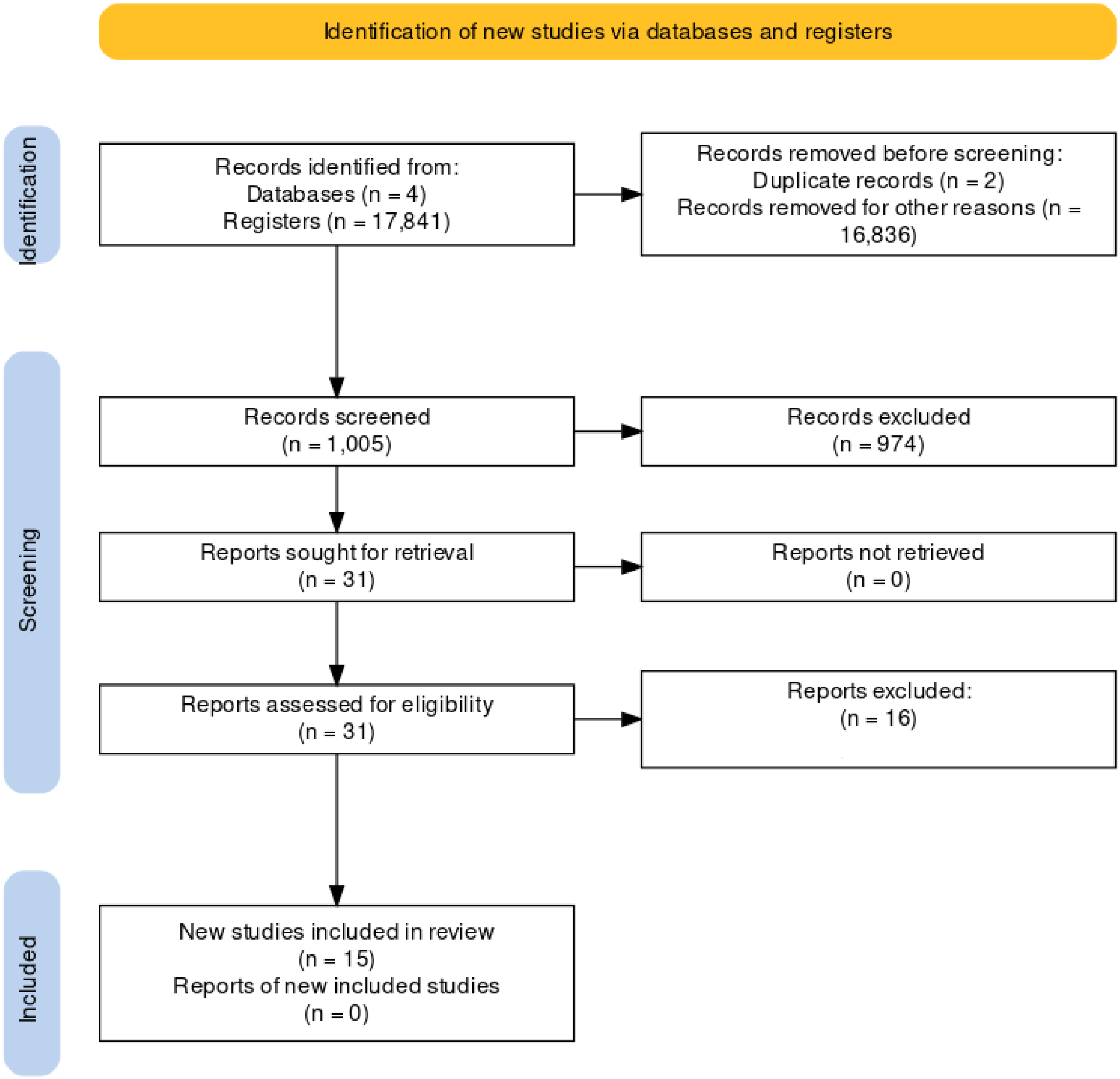


Table 1. Search results

| Database | Results, n |
|---|---|
| Pubmed | 709 |
| Google scholar | 16600 |
| ACM Digital Library | 486 |
| IEEE xplore | 46 |

## Bibliographic data from primary studies

The bibliographic data extracted from each study were author name, year, country. Table 2 shows the bibliographic data.

Table 2. Bibliographic data of the primary papers

| Study | Year | Country |
|---|---|---|
| Essery et al. [38] | 2016 | United Kingdom |
| Collado-Mateo et al. [39] | 2021 | Spain |
| Farrance et al. [40] | 2016 | United Kingdom |
| Rodrigues et al. [41] | 2016 | Canada |
| Room et al. [42] | 2017 | United Kingdom |
| Jordan et al. [43] | 2010 | United Kingdom |
| Picorelli et al. [44] | 2014 | Australia |
| Deka et al. [45] | 2016 | USA |
| Vseteckova et al. [46] | 2018 | United Kingdom |
| Dobson et al. [47] | 2016 | Australia |
| Holt et al. [48] | 2020 | Canada |
| Walker et al. [49] | 2020 | Australia |
| Rodrigues et al. [50] | 2018 | Portugal |
| de Lacy-Vawdon et al. [51] | 2018 | Australia |
| Nicolson et al. [52] | 2017 | Australia |
