# Supplementary material for: Improving Adherence to Physical Therapy in the Development of Serious Games: Conceptual Framework Design Study
Source: JMIR Form Res. 2023 Nov 10;7:e39838. doi: 10.2196/39838 (PMC10674146; doi:10.2196/39838)
Supplement: Multimedia Appendix 2 [file formative_v7i1e39838_app2.pdf]

Multimedia Appendix 2

Key adherence factors identified.

Author	Key adherence factors
Essery et al. [38]	Motivation<br>Perception of therapy<br>Self-Efficacy<br>Perceptions of illness, condition, or injury<br>Formal support (healthcare professional)<br>Natural social support (family and friends)<br>Feedback
Collado-Mateo et al. [39]	Motivation<br>Perception of therapy<br>Self-Efficacy<br>Initial exploration<br>Exercise history<br>Formal support (healthcare professional)<br>Natural social support (family and friends)<br>Social support by the community<br>Personalized exercise program<br>The use of technology<br>Feedback<br>Available Progress Information and Monitoring
Farrance et al. [40]	Motivation<br>Perception of therapy<br>Initial exploration<br>Instructor behaviours (support by healthcare professional)<br>Social connectedness (Social support by the community)<br>Feedback<br>Accessibility
Rodrigues et al. [41]	Motivation<br>Perception of therapy<br>Formal support (healthcare professional)<br>Opportunity for social interactions<br>Flexible program time and modification to exercise plan<br>Feedback<br>Accessibility (Location in a secure, pleasant, or natural environment)
Room et al. [42]	Motivation<br>Self-Efficacy<br>Motivation and intention<br>Social support<br>Feedback
Jordan et al. [43]	Motivation

	Self-Efficacy<br>Social support<br>Personalized exercise program<br>Measure of exercise performance<br>Feedback<br>Accessibility
Picorelli et al. [44]	Motivation<br>Perception of therapy<br>Self-Efficacy<br>Social support<br>Personalized exercise program<br>Feedback<br>Accessibility
Deka et al. [45]	Motivation<br>Self-Efficacy<br>Formal support (healthcare professional)<br>Group support<br>Supervision of exercise<br>Feedback<br>Accessibility<br>Exercise equipment
Vseteckova et al. [46]	Motivation<br>Perception of therapy<br>Self-Efficacy<br>Initial exploration<br>Social support<br>Feedback<br>Accessibility
Dobson et al. [47]	Motivation<br>Perception of therapy<br>Self-Efficacy<br>Exercise history<br>Social support<br>Feedback<br>Environmental Context and Resources
Holt et al. [48]	Motivation<br>Perception of therapy<br>Initial exploration<br>Exercise history<br>Formal support (healthcare professional)<br>Natural social support (family and friends)<br>Social support by the community<br>Personalized exercise program<br>Feedback<br>Accessibility

	Physical environment<br>Program details<br>Diary keeping<br>Reinforcement
Walker et al. [49]	Motivation<br>Perception of therapy<br>Self-Efficacy<br>Initial exploration<br>Exercise history<br>Formal support (healthcare professional)<br>Natural social support (family and friends)<br>Social support by the community<br>Personalized exercise program<br>Assessment of progress<br>Feedback<br>Exercise equipment<br>Progress changeability<br>Acceptance<br>Comfort and convenience
Rodrigues et al. [50]	Motivation<br>Formal support (healthcare professional)<br>Feedback
de Lacy-Vawdon et al. [51]	Motivation<br>Self-Efficacy<br>Social support<br>Personalized exercise program<br>Feedback<br>Progress changeability
Nicolson et al. [52]	Motivation<br>Perception of therapy<br>Self-Efficacy<br>Formal support (healthcare professional)<br>Personalized exercise program<br>Feedback
