# Supplementary material for: Improving Adherence to Physical Therapy in the Development of Serious Games: Conceptual Framework Design Study
Source: JMIR Form Res. 2023 Nov 10;7:e39838. doi: 10.2196/39838 (PMC10674146; doi:10.2196/39838)
Supplement: Multimedia Appendix 3 [file formative_v7i1e39838_app3.pdf]

Multimedia appendix 3

Concept integration.

In compiling the results, several concepts were similar enough to be combined, for example, comfort and convenience, use of exercise equipment, and physical environment are part of accessibility; the ability to modify progress is part of a personalized exercise program. The table shows concept integration, where concepts on the same row are similar in meaning.

Key adherence factor	Concept integration
Motivation	Motivation
Perception of therapy	Perception of therapy
Self-efficacy	Self-efficacy
Initial exploration<br>Exercise history<br>Perceptions of illness, condition, or injury	Initial exploration and exercise history
Formal support (healthcare professional)<br>Natural social support (family and friends)<br>Social support by the community<br>Instructor behaviours (support by healthcare professional)<br>Social connectedness<br>Group support<br>Opportunity for social interactions	Social support
Accessibility<br>Exercise equipment<br>Physical environment<br>Comfort and convenience	Accessibility
Personalized exercise program<br>Progress changeability<br>Flexible program time and modification to exercise plan	Adaptivity
Feedback<br>Reinforcement	Feedback
Use of technology to record the frequency, duration, and time of the exercises<br>Measure of exercise performance<br>Supervision of exercise<br>Diary keeping<br>Available Progress Information and Monitoring<br>Assessment of progress	Available progress information
